# A Lateral Flow Strip Based Aptasensor for Detection of Ochratoxin A in Corn Samples

**DOI:** 10.3390/molecules23020291

**Published:** 2018-01-31

**Authors:** Guilan Zhang, Chao Zhu, Yafei Huang, Jiao Yan, Ailiang Chen

**Affiliations:** 1 Key Laboratory of Agro-Product Quality and Safety, Institute of Quality Standards and Testing Technology for Agro-Products, Chinese Academy of Agricultural Sciences, Beijing 100081, China; zhangguilan2011@163.com (G.Z.); ndytzhuchao@126.com (C.Z.); yaifei@126.com (Y.H.); yanjiao9999@gmail.com (J.Y.); 2 College of Food Science and Technology, Hainan University, Haikou 570228, China

**Keywords:** aptamer, lateral flow strip, detection, Ochratoxin A, corn samples

## Abstract

Ochratoxin A (OTA) is a mycotoxin identified as a contaminant in grains and wine throughout the world, and convenient, rapid and sensitive detection methods for OTA have been a long-felt need for food safety monitoring. Herein, we presented a new competitive format based lateral flow strip fluorescent aptasensor for one-step determination of OTA in corn samples. Briefly, biotin-cDNA was immobilized on the surface of a nitrocellulose filter on the test line. Without OTA, Cy5-labeled aptamer combined with complementary strands formed a stable double helix. In the presence of OTA, however, the Cy5-aptamer/OTA complexes were generated, and therefore less free aptamer was captured in the test zone, leading to an obvious decrease in fluorescent signals on the test line. The test strip showed an excellent linear relationship in the range from 1 ng·mL^−1^ to 1000 ng·mL^−1^ with the LOD of 0.40 ng·mL^−1^, IC_15_ value of 3.46 ng·mL^−1^ and recoveries from 96.4% to 104.67% in spiked corn samples. Thus, the strip sensor developed in this study is an acceptable alternative for rapid detection of the OTA level in grain samples.

## 1. Introduction

Ochratoxin A (OTA), a mycotoxin produced from species of fungi including *Penicillium verrucosum, Aspergillus ochraceus, Aspergillus carbonarius and Aspergillus niger* [1,2], could be easily found in many improperly stored food and agricultural products [3] such as coffee [4], beer [5] and wine [6,7]. Due to its chemical stability during food and feed processing, OTA may reside in foodstuff, spices and animal feed [8,9,10,11]. In addition, OTA is a nephrotoxic toxin [12] with strong carcinogenic effects on rodents [13]. OTA has been proven to be teratogenic, embryotoxic, genotoxic, neurotoxic and immunosuppressive [14,15]. Due to the extreme toxicity of OTA, many countries and organizations have set guidelines and recommendations for the maximum residue level (MRL) [16], for example, 5 μg·kg^−1^ and 10 μg·kg^−1^ of OTA are considered acceptable in cereals by the World Health Organization and European Union and China, respectively.

Rapid and sensitive detection of OTA in foodstuff has become greatly important for food safety control. OTA detection in food is usually performed using instruments, such as high-performance liquid chromatography (HPLC) [17], ultra-performance liquid chromatography with fluorescence detector (UPLC-FLD) [18], ultra-fast liquid chromatography coupled with electrospray ionization tandem mass spectrometry (UFLC-ESI-MS/MS) [19], gas chromatography-mass spectrometry (GC-MS) [20] and liquid chromatography-mass spectrometry (LC-MS) [21]. Although they have good accuracy and reproducibility, they cannot be widely used for rapid onsite detection because of expensive equipment, demand for highly qualified personnel and costly and time-consuming pretreatment.

To overcome the limitations of chromatographic analysis and immunoassay methods such as enzyme-linked immunosorbent assay (ELISA) [22], immunochromatographic assay (ICGA) [23] and electrochemical biosensors [24] have been developed. However, these screening methods rely heavily on antibodies with the following disadvantages: time-consuming production of antibodies, poor stability and high sensitivity to pH and temperature, modification difficulty and high cost, high immunogenecity and low bioavailability in in vivo applications. Therefore, seeking substitutes for antibodies involved in the detection methods is tremendously important.

Aptamers [25,26] are single-stranded oligonucleotides selected from a DNA or RNA library in vitro by a standard process named systematic evolution of ligands by exponential enrichment (SELEX), which can bind to target molecules with high affinity and specificity by folding into specific three-dimensional structures. Aptamer has high specificity, chemical stability, long shelf-life, chemical synthesis and modification simplicity, high biodegradation stability and low vulnerability to denaturation [27], and therefore aptamers have so far been widely applied to drug delivery [28], disease diagnosis [29], targeted therapy [30], cell imaging [31] and biotechnology for detection of proteins [32], metal ions [33], antibiotics [34], pesticides [35], and even cells [36], bacteria [37] and others [38]. Aptamer-based rapid detection methods include colorimetry, fluorescence, matrix-assisted laser desorption/ionization mass spectrometry (MALDI-MS), electrogenerated chemiluminescence (ECL), surface plasmon resonance (SPR), polarization, western blotting and electrochemistry [39]. And aptamer-based biosensors are considered a promising molecular diagnostic approach in the future. At present, there are many methods based on aptamer for detection of OTA, such as electrochemical [40,41,42], colorimetry [43], fluorescence [44,45], chemiluminescence [46] and up-converting luminescence [47]. Although they have good sensitivity and specificity, they cannot be used for onsite one-step detection with the following disadvantages: a small linear range in tests, complex synthesis and immobilization process of nanoparticles or sensor.

Lateral flow assay (LFA) [48] has been used for detection of clinical and non-clinical analytes with the “one-step” and “onsite” advantages. Lateral flow strip biosensor (LFB) [49] based aptamer has been employed to follow biorecognition events, such as detecting Escherichia coli O157:H7 [50], IgE [51], thrombin [52] and ATP [53] with unique characteristics of sensitivity, specificity and operation simplicity. And complex and expensive instrumentation are not needed for the method. For OTA, the LFB based aptamer is mainly visual based GNP-antibody [54,55] and GNP-aptamer (gold nanoparticles or GNPs) [56,57]. But, these strips are mostly used for qualitative [58,59] or half quantitative detection of OTA [60,61] without specific detection range or with a small linear range in testing. And, they are mostly used to detect OTA in wine and grape must [62]. So, the purpose of this study was to establish a stable, rapid, simple and accurate lateral flow strip biosensor to quantitatively detect OTA in corn samples with a large linear range for testing.

Here, we described a novel aptamer-based lateral flow fluorescent strip for rapid detection of OTA. The fluorescence intensity of this lateral flow strip biosensor exhibited a good linear relationship with the OTA concentration ranging from 1 ng·mL^−1^ to 1000 ng·mL^−1^ with the LOD of 0.40 ng·mL^−1^ and IC_15_ value of 3.46 ng·mL^−1^. The developed strip was also applied for detection of OTA in corn samples, and a satisfactory result could be obtained. Therefore, it is a promising technique for practical use in mycotoxin screening for agricultural products and food.

## 2. Materials and Methods

### 2.1. Reagents and Apparatus

OTA, aflatoxin B_1_ (AFB_1_), aflatoxin G_1_ (AFG_1_), aflatoxin M_1_ (AFM_1_), zearalenone (ZEN), fumonisin B_1_ (FB_1_) and tris(hydroxymethyl)aminomethane (Tris) (purity > 99%) were purchased from Sigma-Aldrich (St. Louis, MO, USA). Poly(ethylene glycol) with average M.W. 20,000 (PEG 20,000) was obtained from J&K Scientific Ltd. (Beijing, China). Polyoxyethylene sorbitan monolaurate (Tween 20) was from Tokyo Chemical Industry Co., Ltd. (Shanghai, China). Absorbent pad (H5076), sample pad (GL-b04), nitrocellulose filter (Sartorius CN140) and backing pad were acquired from Shanghai Jieyi Biotechnology Co., Ltd. (Shanghai, China).

Ultrapure water used throughout all experiments was purified by a Milli-Q system (Millipore, Bedford, MA, USA) with the resistivity of 18.2 MΩ cm. Strip signals were scanned using an ESLF10-SO-1010 Lateral Flow Studio V3.3.8 (Qiagen, Germany). Every test strip was prepared with a 3050 dispensing platform and KM-3100 Cutter (BioDot, Irvine, CA, USA).

All other reagents, purchased from Beijing Chemical Reagent Company (Beijing, China), were of analytical grade and used without further purification or treatment unless otherwise specified.

Buffer A, B, C and D components of Tris-HCl were all 10 mM Tris, 120 mM NaCl, 20 mM CaCl_2_, 5 mM KCl, 0.1% Tween-20 (*v*/*v*), 1% PEG 20,000 (*m*/*v*), 2% sucrose (*m*/*v*) and 5% methanol, and at pH 7.4, 8.0, 8.5 and 9.0, respectively. Buffer E was 10 mM sodium citrate-hydrochloric acid solution, 200 mM NaCl, 20 mM CaCl_2_, 20 mM MgCl, 0.1% Tween-20 (*v*/*v*), 1% PEG 20,000 (*m*/*v*), 2% sucrose (*m*/*v*) and 5% methanol. Buffer F consisted of 100 mL 10 mM PB solution, 0.5 g PEG 20,000, 1 g sucrose, 0.1 mL Tween-20, 0.02 g MgSO_4_ and 0.05 g (NH_4_)_2_SO_4_.

All DNA sequences were ordered from Sangon Biotech (Shanghai, China), and lyophilized powder was dissolved in ultrapure water and stored at 4 °C before use. DNA sequences used in this research were as follows (from 5′ to 3′): 

OTA aptamer [63]:

5′-cy5-(CH_2_)_6_-aaa-aaa-aaa-aaa-aaa-aaa-gat-cgg-gtg-tgg-gtg-gcg-taa-agg-gag-cat-cgg-aca-3′
cDNA: 5′-biotin-(CH_2_)_6_-aaa-aaa-tgt-ccg-atg-ctc-cct-tta-cgc-cac-cca-cac-ccg-atc-3′
probe 2: 5′-biotin-(CH_2_)_6_-ttt-ttt-ttt-ttt-ttt-ttt-3′

### 2.2. Preparation of Streptavidin-Biotin-DNA Probe Conjugates

To immobilize test and control probes on a nitrocellulose (NC) membrane, biotin-modified cDNA and probe 2 were combined with streptavidin to form streptavidin-biotin-DNA conjugate via a biotin-streptavidin affinity reaction. Briefly, streptavidin was dissolved into 0.01 M phosphate-buffered saline (PBS) solution (pH 7.4) at 1 mg·mL^−1^, and 5 μL of 1 mg·mL^−1^ streptavidin solution and 35 μL of 3 μM biotin-cDNA solution were incubated at 25 °C for 2 h. Probe 2 was similarly constructed as described above with probe 2 used instead of cDNA. Then, the streptavidin-biotin-DNA probe conjugate was stored at 4 °C and used for subsequent fabrication of the control and test lines on the strips.

### 2.3. Fabrication of Aptamer-Based Strips

**Figure 1:**
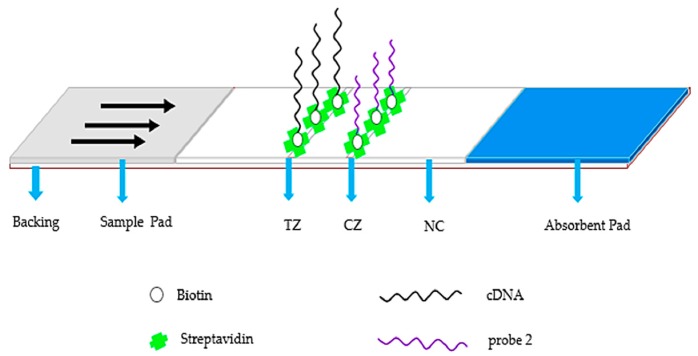
Schematic illustration of the configuration of a conventional strip biosensor. SA–biotin–­cDNA and SA–­biotin–­probe 2 dispersed in the Test Zone (TZ) and Control Zone (CZ), respectively.

The preparation of the strips is shown in Figure 1. NC membrane, sample pad and absorbent pad were all pasted onto a plastic backing plate and overlapped 2 mm with each other in sequence. Streptavidin-cDNA and streptavidin-DNA probe 2 conjugates were dispersed at an interval of 5 mm on the membrane as the test line and control line, respectively. The two lines were positioned at a 4 mm interval. The plate was dried at 37 °C for 30 min and then cut into 4.0 mm wide strips using the programmable strip cutter KM-3100. They were then put into valve bags, which were stored at 4 °C for future detection tests.

### 2.4. OTA Detection

In our test, 50 μL of sample solution was mixed with 5 μL of Cy5-labeled aptamer in an *Eppendorf* (EP) tube and incubated at room temperature for 10 min. Then, each 50 μL portion of the mixture was dripped onto the sample pad of a lateral flow test strip, allowing all liquid to migrate along the strip for absorption. 10 min later, the test strip was analyzed by Lateral Flow Studio so that the fluorescent intensities on both the T line and C line were acquired in the integrated area. To minimize system and random errors in the experiment and increase the signal to noise ratio (SNR), the ratio of the T/C fluorescence intensity was calculated as an evaluation indicator for drawing a standard curve for quantification of the OTA concentrations in the samples. A fluorescence decrease in the T/C value was detected each time OTA was applied.

### 2.5. Strip Sensitivity and Specificity Detection

To detect the sensitivity of the strip, 5 μL of 0.05 μM Cy5-labeled aptamer was mixed with 50 μL of OTA standard solution at different concentrations (0, 1, 3, 10, 30, 100, 300 and 1000 ng·mL^−1^) by diluting OTA stock solution (100 μg·mL^−1^ in methanol) with Tris-HCl buffer. The reaction solution was stirred and incubated at room temperature for 10 min, and then each 50 μL portion was pipetted onto the sample pad. After 10 min, the fluorescence intensities at both the test line and control line were measured by Lateral Flow Studio. The procedure was repeated to estimate the selectivity of the method by replacing OTA with other mycotoxins.

### 2.6. Applications of Test Strips to Samples

Accurately weighed 2 g of ground corn powder sample was extracted with 10 mL of methanol-water (70:30, *v*/*v*) by violently shaking for 5 min, followed by sonication in an ultrasound bath for further 30 min at room temperature and centrifugation at 10,000 rpm for 10 min. The supernatant was dried by nitrogen blowing and then dissolved using 10 mL pH 8.5 Tris-HCl to minimize the influence of the complex matrix and methanol. After that, corn sample extract solution was spiked with OTA stock solution (100 μg·mL^−1^ in methanol) at a series of concentrations of 3, 10, and 30 ng·mL^−1^. Then, 50 μL portion of each sample extract solution containing OTA standard solution was applied to the aptamer-based lateral flow strip for detection of OTA, which was repeated three times. The test results were compared with the actual concentrations, and finally the average recovery was calculated.

## 3. Results and Discussion

### 3.1. Concept and Aptasensor Mechanism

**Figure 2:**
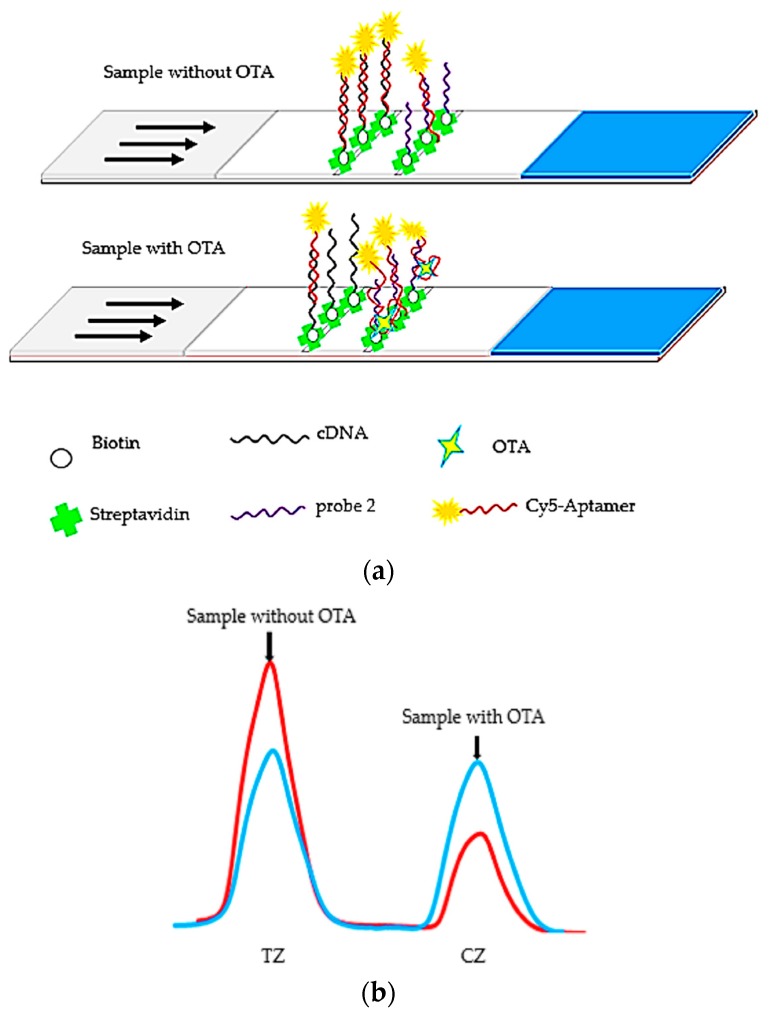
Schematic illustration of competitive aptamer-based lateral flow strip biosensors used for OTA quantification. (**a**) Strips presented before and after OTA application; (**b**) The changes in the fluorescence intensities in the control and test line regions quantified by estimating the intensities of the bands.

The proposed aptamer-based lateral flow fluorescent strip for OTA detection was fabricated, as shown in Figure 1. In the schematic diagram (Figure 2), the LFB was prepared based on the competitive reaction between cDNA (test line) and Cy5-labeled aptamer, indicating that the fluorescence intensity ratio of the T line to C line was inversely proportional to the OTA concentration in the samples. When the sample solution without OTA was dropped onto the sample pad, the mixture migrated to the test zone with capillary dynamic and Cy5-labeled aptamer was captured largely by cDNA to form a stable double helix, resulting in strong fluorescence intensities in the control and test zones, respectively. In the presence of OTA, however, OTA would combine with the aptamer probe, reducing the amount of Cy5-labeled aptamer, which could hybridize to cDNA on the test line, and therefore the fluorescence intensity became weaker. In other words, the more aptamer-OTA complexes existed in the solution, the less free Cy5-labeled aptamer would be captured on the test line. And no matter whether OTA was present in the detection solution, aptamer probes would definitely hybridize with probe 2 on the control line, ensuring the detection validity. If not, the detection may become invalid.

### 3.2. Optimization of the Aptamer Concentration

**Figure 3:**
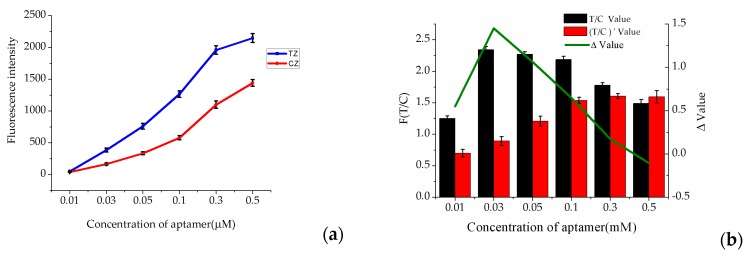
Optimization of the aptamer concentration. (**a**) The trend of the fluorescence intensities (Test Zone (TZ) and Control Zone (CZ)) with the increase of the aptamer concentration; (**b**) The variation in the T/C value before (T/C Value) and after ((T/C)’ Value) the solution was spiked with 30 ng·mL^−1^ OTA (*n* = 3).

The aptamer concentration was directly associated with the detection limit of aptamer-based lateral flow strip. To determine the optimum concentration of aptamer, 50 μL of 10 mM Tris-HCl buffer was mixed with 5 μL of Cy5-labeled aptamer at different concentrations (0.01, 0.03, 0.05, 0.1, 0.3 and 0.5 μM) in ultrapure water, with dramatic differences between 0 ng·mL^−1^ and 30 ng·mL^−1^ OTA standard solution. According to the results (Figure 3), the fluorescence intensities (TZ and CZ) were gradually enhanced with the increase of the aptamer concentration, but the T/C value showed a trend of decline from 0.03 to 0.5 μM. Moreover, the most varied T/C value before (T/C Value) and after ((T/C)’ Value) the solution was spiked with 30 ng·mL^−1^ OTA was 0.03 μM. So, 0.03 μM aptamer was the best choice for the following experiments with the maximum response value, highest sensitivity and minimum error.

### 3.3. Effect of pH Value of the Running Buffer on the Strip Sensor Performance

**Figure 4:**
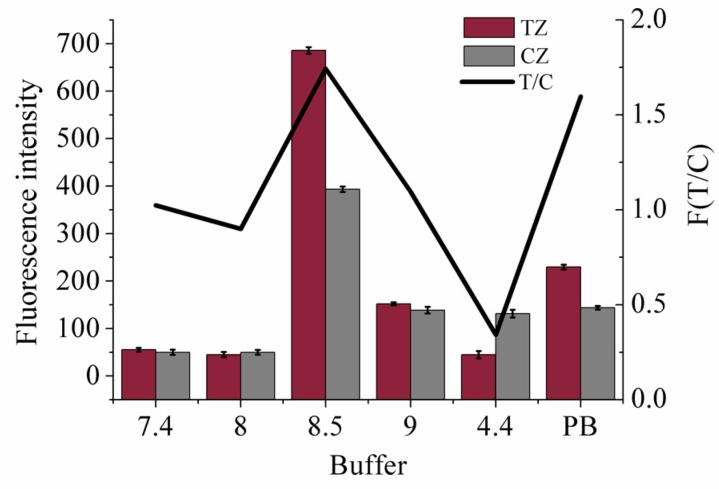
Running buffer optimization. Fluorescence intensity values of TZ, CZ and T/C with different kinds of buffer applied (*n* = 3).

The pH value and ion components of the running buffer have a significant influence on oligonucleotide chain hybridization and combination stability between aptamer and the target, so that the optimal buffer is critical for the performance of strip sensors. Therefore, the pH dependence of the performance of strip biosensors for OTA determination was tested. An expected buffer should ensure that the fluorescent signals of the C line can be scanned in any case. To prevent nonspecific binding and achieve adequate hybridization reaction, the maximum response values of the T&C lines on the membrane were acquired with the minimum error of the measured value. The reason for the maximum signal at pH 8.5 and low signal at pH 9 may be that the electric charge and H+ affected the secondary structure of aptamer and hybridization efficiency. In addition, the active substances on the surface of NC membrane influenced the flow and hybridization of DNA. With multiple factors considered, pH 8.5 was the optimal reaction conditions. After investigation (Figure 4), buffer C was determined as the running buffer.

### 3.4. Sensitivity and Specificity

**Figure 5:**
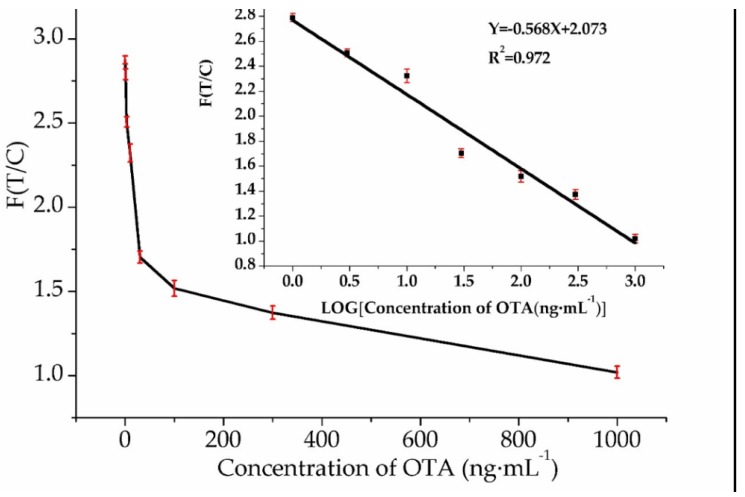
Calibration curve for OTA detection. The inset shows the linear relationship between the fluorescence intensity ratio of the TZ to CZ and the OTA concentration within the range of 1–1000 ng·mL^−1^. Error bars represent the standard deviations of three replicates (*n* = 3).

To measure the sensitivity of the developed method, OTA standard solutions at various concentrations of 0, 1, 3, 10, 30, 100, 300 and 1000 ng·mL^−1^ were prepared by diluting OTA stock solution (100 μg·mL^−1^ in methanol) with the running buffer. A total of 10 μL aptamer and 50 μL OTA solution were accurately pipetted in an EP tube and incubated at room temperature for 10 min. Then, each 50 μL portion of the mixture solution was pipetted onto the sample pad of the strip, and after another 10 min, the fluorescence intensities at both the test line and control line were obtained using Lateral Flow Studio scan strip. The detection was performed in three repeats for each concentration. The results in Figure 5 showed that the strip had high fluorescence values on both the test and control lines, and the ratio of the T/C fluorescence intensity decreased with the OTA concentration increase from 1 ng·mL^−1^ to 1000 ng·mL^−1^ (R^2^ = 0.972). The limit of detection (LOD) was determined to be 0.40 ng·mL^−1^ according to three times the standard deviation of the blank/slope. And the fifteen percent maximal inhibitory concentration (IC_15_) was 3.46 ng·mL^−1^.

**Figure 6:**
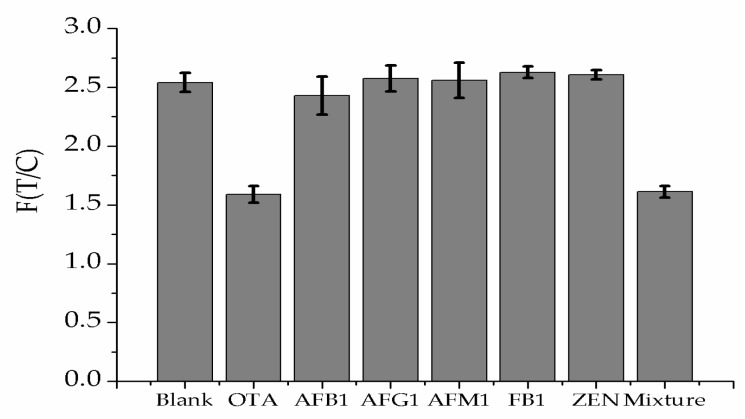
Specificity of the method. Fluorescence intensity ratio of the TZ to CZ with OTA and other mycotoxins (*n* = 3).

The selectivity of LFB was tested using various mycotoxins for this study. The 30 ng·mL^−1^ OTA standard and other similar mycotoxin standards including aflatoxin B_1_ (AFB_1_), aflatoxin G_1_ (AFG_1_), aflatoxin M_1_ (AFM_1_), zearalenone (ZEN) and fumonisin B_1_ (FB_1_) were tested by the optimized lateral flow strips. As shown in Figure 6, the other mycotoxins displayed a basic consistent level of the fluorescence intensity ratio of the test line to the control line compared with the blank solution, only the OTA-spiked corn sample gave a significant reduction, indicating good specificity and selectivity of the strip for OTA.

### 3.5. Practical Sample Analysis

To evaluate the practicability and accuracy of the prepared aptamer based lateral flow strip, corn samples added with OTA standard solutions at a series of concentrations were analyzed by the lateral flow strip. The samples at each concentration were repeated 3 times and the results were shown in Table 1. The recovery test showed that the fluorescent strip aptasensor exhibited a good recovery rate within the range from 96.40% to 104.67%, and our assay had excellent accuracy and stability in detecting OTA in real samples. To further verify the superior performance of the prepared lateral flow strip biosensors, the detection range, detection limit and sample matrixes were compared with those of other methods reported in the literatures. The detailed results were listed in Table 2. We could see that other strips in the published literatures mostly used for qualitative detection or half quantitative detection of OTA with a small linear range in testing wine and grape must samples. It can also be seen that the proposed sensor had a differently wide linear range and low detection limit, making it a trend to screen test samples with the advantages of easy sample treatment, short detection time, low cost and low requirements on professional skills.

## 4. Conclusions

In this study, simple, specific, sensitive, and easily applied Cy5-labeled aptamer based lateral flow strip biosensors in the competitive format were developed for detection of OTA. Under the optimal conditions, quantitative detection of OTA using a portable strip reader exhibited a linear relationship between the ratio of the fluorescence intensities on the test line to the control line and the logarithm of the OTA concentration in the range of 1–1000 ng·mL^−1^ with the detection limit of 0.40 ng·mL^−1^ and IC_15_ value of 3.46 ng·mL^−1^. And the entire detection process could be accomplished within 20 min. As the aptamers were not special, the strip would be applicable to any rapid onsite detection of OTA in corn samples, and the immunochromatographic strip test could be used for rapid qualitative and quantitative screening of OTA contamination in grain and foodstuff samples.

## Figures and Tables

**Table 1:** Detection results of the OTA levels in spiked corn samples (*n* = 3).

Spiked (ng·mL^−1^)	Detected (ng·mL^−1^)	Recovery (%)	CV (%)
3.00	3.14 ± 0.47	104.67	6.40
10.00	9.64 ± 1.15	96.40	4.80
30.00	29.17 ± 2.73	97.23	5.10

**Table 2:** Comparison of the prepared sensor with other reported strip methods for OTA detection.

Method	Range (ng·mL^−1^)	LOD (ng·mL^−1^)	Sample	Reference
Antibody-strip	-	10	PBS buffer	[58]
Antibody-strip	1.00–6.00	0.77	cereal	[60]
Antibody-strip	-	10	corn, wheat	[54]
Antibody-strip	-	1	wine, grape must	[55]
Antibody-strip	-	0.9	wine, grape must	[62]
Antibody-strip	-	0.5	wed wine	[59]
Apatamer-strip	0.5–2.5	0.18	red wine	[56]
Apatamer-strip	0.5–25	0.5	*Astragalus membranaceus*	[61]
Aptamer-strip	0.10–10.00	1.90	-	[57]
Aptamer-strip	1.00–1000.00	0.40	corn	Present
